# Genetic and environmental components of female depression as a function of the severity of the disorder

**DOI:** 10.1002/brb3.519

**Published:** 2016-08-12

**Authors:** James S. M. Rusby, Fiona Tasker, Lynn Cherkas

**Affiliations:** ^1^ Department of Psychological SciencesBirkbeck University of LondonLondonUK; ^2^ Department of Twin Research and Genetic EpidemiologyKing's College LondonLondonUK

**Keywords:** additive and dominance estimates, homozygotic and heterozygotic alleles, major depression, mood disorders, twin studies

## Abstract

**Background:**

Both clinical care and genome‐wide studies need to account for levels of severity in the etiology of depression. The purpose of the study is to estimate the genetic and environmental components of female depression as a function of the severity of the disorder.

**Methods:**

A genetic and environmental model analysis of depression incidence was made using the IOP Depression Severity Measure (IDSM). Details of lifetime depression incidence were obtained by questionnaire from twins on the DTR registry. Data from 1449 matched female twin pairs in the age range 19–85 years in four ordinal categories of increasing severity were employed in the analysis.

**Results:**

Estimates of additive and dominance genetic components of 27% and 25% were found when all three levels of depression were included, and near zero and 33% when the recurrent/severe level was excluded. Shared environmental effects were not significant in either case, but the estimate for random environmental effects was greater when the severe level was excluded.

**Conclusions:**

These results suggest that the incidence of severe depression is associated with homozygotic alleles and the less severe with heterozygotic alleles. This is in accord with the finding that the hereditary component of severe depression is relatively high and that milder forms are more dependent on life‐time environmental factors. Such conclusions have clinical implications for the diagnosis and treatment of the disorder by practicing psychiatrists. They also lead to the importance of focusing future genome‐wide and linkage studies on those females with severe levels of depression if progress in identifying genetic risk loci is to be made.

## 1 Introduction

Clinical depression is one of the most common psychiatric disorders, particularly in Europe and America with a lifetime prevalence rate between 10% and 20% where it is a major cause of morbidity and consequent disability (Demyttenaere et al., 2014). As a result, a considerable research effort has been made over the past few years to try to gain some insight into the etiology of this disorder. A meta‐analysis of six high‐quality twin studies including both sexes have estimated the genetic component to be about 37% and this figure has been confirmed by a large study from Sweden (Kendler, Gatz, Gardner, & Pedersen, 2006; Sullivan, Neale, & Kendler, 2000). These studies have also concluded that the figure may be higher for those with severe or recurrent forms of the illness. The shared or family environmental components have been found to be of little significance, but lifetime environmental factors are important. It is well known that women are at a greater risk of depression and there is increasing evidence that this is partly due to the higher genetic component found for female depression, where research evidence estimates genetic components of some 40%–44% for women and 21%–31% for men (Bierut et al., 1999; Jansson et al., 2004; Kendler, Gardner, Neale, & Prescott, 2001). These substantive results from twin analyses have encouraged international groups of researchers to use both GWAS and linkage studies to try to ascertain which candidate genes might contribute to the etiology of depression. However, as Flint and Kendler (2014) conclude from their extensive review of these complex and ambitious investigations, many articles have been generated but no robust findings or positive replications made. In addition, parallel research progress based on the original monoamine hypothesis has been limited now that certain aspects of its explanatory performance have been found to be inadequate (Hirschfeld, 2000; Krishnan & Nestler, 2008). Flint and Kendler believe that the mega‐analysis of GWAS studies reported by Ripke et al. (2013) totaling some 9,000 cases of clinical depression that failed to find robust evidence for loci that exceeded genome‐wide significance levels implies that genetic variance may be due to the joint effect of large numbers of loci of small effect. This suggests that clinical depression may be diverse in origin and so unlikely to be dichotomous in outcome.

These past, inconclusive, GWAS and linkage results based on the dichotic selection of control and incidence groups, and in which sex and age are usually pooled, will need to be better focused in future if progress is to be made in locating the genetic loci contributing to the etiology of depression. As a contribution in this direction, the analysis described in the present paper is limited to female cases to investigate whether genetic and environmental variance components of depression may vary as a function of the severity of this disorder.

Any analysis such as this which attempts to clarify the etiology of clinical depression as a function of the severity of the disorder will also be of value to practicing clinicians as they try to assess the possible origins of individual cases and the treatment required. For those who may be unfamiliar with such genetic analyses, it is worth clarifying and defining the terms used. In this study, the classic genetic model is employed in a structural analysis to estimate both the genetic and environmental variance components related to the phenotype, depression incidence. The genetic components may be additive or dominant in form. If additive, the responsible genes have two dichotic alleles which are identical or homozygotic. If dominant, then one allele is responsible for the effect, and the other, recessive, allele does not contribute, and the gene is heterozygotic. Dominance may be complete, in which case the effect on the phenotype is the same as the additive effect, or partial, leading to an intermediate dosage. Hereditary influence is largely due to the additive component. The variance of the environmental components estimated by the analysis is also in two forms: the first is due to shared or family factors and the second to random factors during development and in adult life. In practice, the effect on the phenotype is very likely to be due to the aggregated influence of a number of contributing genetic loci and also on a range of environmental effects. The classic structural genetic analysis has been shown to be valid for such multiple effects (Neale & Cardon, 1992).

## 2 Method

### 2.1 Subjects

Subjects were volunteer female monozygotic (MZ) and dizygotic (DZ) twins enrolled in the TwinsUK Adult Twin Registry held in the Department of Twin Research (DTR) at St Thomas’ Hospital, London (Spector & Williams, 2006). All twins were recruited through national media campaigns and from other twin registers. The twins in the registry are not selected for any particular trait and they volunteer to take part in studies that cover a wide range of traits and common medical conditions. The study was approved by the St Thomas’ Hospital Research Ethics Committee and all twins in the study provided their consent. Twins from this registry have been shown to be comparable to the age‐matched general population of singletons for a broad variety of medical and behavioral traits (Andrew, Hart, Sneider, & De Lange, 2001).

In 2006, the IOP Depression Severity Measure (IDSM) was included in the annual questionnaire sent to 8,990 registered twins by the Department of Twin Research. A total of 5,097 replied giving a response rate of 57% of which 91% were females (*N*_f_ = 4,638). MZ and DZ twins accounted for 57% and 42% of the respondents and in 1% zygosity was undetermined. Age ranged from 19 to 85 years. After including only the paired female twins, reared together, of known zygosity and white ethnicity, 1,740 twin pairs were available (*N*_F_ = 3,480, or 75%), 973 monozygotic, and 767 dizygotic twin pairs. In order to equalize the numbers of MZ and DZ pairs, this number was then reduced to 1,449 twin pairs for the model genetic analysis.

### 2.2 Assessment of depression

The IDSM depression measure used was developed at the Institute of Psychiatry (IOP) and assessed for content validity by Professors David Collier and Anne Farmer of IOP (see Appendix 1). The measure has been employed by Rusby, Harris, and Tasker (2013) to study the association between personal dependency and levels of depression and by Davies et al. ([Link]) to examine the relationship between epigenetic methylation effects and depression. Three levels of depression of increasing severity are assessed by the design. The first or mild depression, is self‐diagnostic and is scored by those who answer ‘yes’ to question 1, but excludes those women who only had depression following the birth of a baby. The second or moderate level, is scored if, in addition, a general practitioner or psychiatrist had diagnosed depression, that is ‘yes’ to question 3 or 4. The final, severe/recurrent, level is scored if the respondent has received defined medication from a general practitioner or psychiatrist, that is ‘yes’ to question 6c, and/or had more than one episode of depression that required some form of treatment, that is ‘yes’ to question 8. In order to avoid any experimenter bias leading to spurious linkage between individual twin pairs scoring was automatically carried out by STATA software using the IDSM depression level scoring algorithm described above. For the genetic analysis, these levels were scored in one of four ordinal categories, from 0, no depression, through to 3, severe/recurrent depression.

### 2.3 Twin analysis

**Figure 1:**
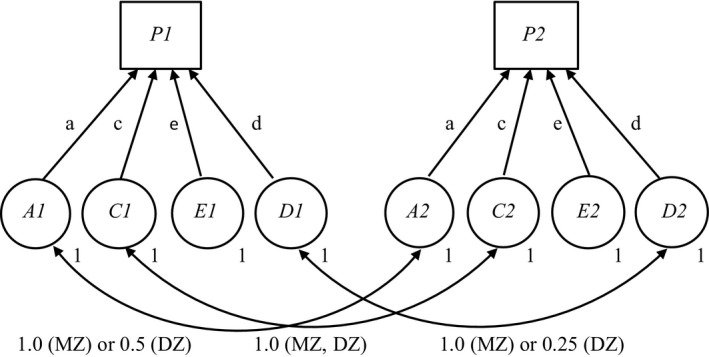
Basic genetic path model for data from monozygotic (MZ) and dizygotic (DZ) twins reared together

The genetic and environmental analysis proceeded from the use of the classic structural path model for data from monozygotic and dizygotic twins reared together (Neale & Cardon, 1992). The layout of this path coefficients model is shown in Fig. 1. In the model, the latent additive genetic factors *A*1 and *A*2, the dominant genetic factors *D*1 and *D*2, the shared environmental factors *C*1 and *C*2, and the random environmental factors *E*1 and *E*2, cause the phenotypes *P*1 and *P*2 in twins 1 and 2. It is not expected that the values of the genetic or environmental effects, a, c, e, and d, as free parameters will vary between twins, and these constraints are included in the running of the analysis. In addition, the variance of the latent factors are standardized to unity. Classical covariance values from genetic theory linking the factors for both types of zygosity are included in the figure.

The EQS structural program employed was run as a two‐group analysis in which the results from each MZ and DZ modeling analysis were combined in order to determine the best‐fitting genetic and environmental estimates (Bentler, 1995). Since the data are categorical with four ordinal levels, arbitrary generalized least squares (AGLS) estimation was used in which the correlations between the depression level scores were estimated by polychoric and polyserial coefficients (Lee, Poon, & Bentler, 1994). The EQS program allows an estimation of the goodness‐of‐fit in the model analysis to be made based on the likelihood ratio χ^2^ between the nested models and observed variance–covariance matrices. This is the method recommended by Neale and Cardon (1992) for obtaining such goodness‐of‐fit criteria.

## 3 Results

As described above, the depression incidence data generated by the IDSM measure was defined by four ordinal categories. In terms of incidence, 63% reported no depression, 8% mild depression, 14% moderate depression, and 15% severe levels of depression. Twin pair correlations were 0.40 and 0.14 for the MZ and DZ pairs, respectively, resulting in a heritability estimate of 52%. If the severe category is omitted, the correlations were 0.18 and 0.06 giving a reduced heritability of 24%.

Two genetic analyses were carried out: in the first, all three levels of depression incidence were included, and in the second, the severe level was omitted and only the mild and moderate levels retained. In order to equalize case numbers for the analyses, the numbers of MZ twin pairs were reduced randomly by SPSS from 973 to 726 to be equal to the number of DZ twin pairs in the first model analysis and from 767 to 520 in the second analysis, giving a total of 1,449 and 1,052 twin pairs, respectively. This follows the recommendation given by Neale and Cardon when a two‐group analysis is run which combines the data from both sets of twin pairs, MZ and DZ (Neale & Cardon, 1992).

The model fitting results obtained are listed in Table 1 for the five nested models evaluated for each depression severity analysis, excluding the ACDE model which is not identified in the classical twin model. In both analyses, the shared environment effect, C, is not significant and the models with the highest probability of fitting the observed covariance are the ADE models. Both of these ADE model are saturated, that is, there are only one set of values for the three parameters for each of the ADE models which will produce expected values of the covariance–variance matrix that match the observed values (Purcell, 2013). In the case of the second analysis, in which the severe level of depression has been excluded, the ADE model needs to be tested against the results for the more parsimonious DE model, which also has a low value of χ^2^, to check if the additive component in the ADE model is significant. The difference in χ^2^ between the models is 2.6 with *df* = 1, giving a probability *p* = .11 which is not significant at the .05 level. This suggests that the more parsimonious DE model is to be preferred since it does not show a reduction in fit and so provides evidence that the additive genetic component in the ADE model is not likely to be significant.

**Table 1:** Model fitting results for female depression

Model (*df*)	Severe level included (1,449 twin pairs)		Severe level excluded (1,052 twin pairs)	
	χ^2^	*p*	χ^2^	*p*
ADE (3)	0.0	1.00	0.00	1.00
ACE (3)	18.6	<.001	>1,000	<.001
AE (4)	18.6	<.001	29.6	<.001
CE (4)	293.6	<.001	546.3	<.001
DE (4)	25.7	<.001	2.6	.27

Table 2 lists the estimated values of the path coefficients with their confidence intervals and the variances of the two conditions analyzed. The total additive, a^2^, plus d^2^, dominant, genetic variance estimated from the analysis which includes the severe category of depression is 52% which falls to 33% due to the dominant variance alone when only the two nonsevere ordinal levels of depression are included. In both cases, the remaining variance is accounted for by random environmental effects including residual error (e^2^ values) and none by shared or family effects (c^2^ values).

**Table 2:** Path coefficients and variance estimates for ADE model from MZ and DZ two‐group analyses

Depression	Path coefficients			Variance estimates		
	a (CI_.95_)	d (CI_.95_)	e (CI_.95_)	a^2^	d^2^	e^2^
With severe	0.52 (0.16)	0.50 (0.18)	0.69 (0.02)	0.27	0.25	0.48
Nonsevere	0	0.58 (0.16)	0.82 (0.09)	0	0.33	0.67

## 4 Discussion

The finding that the total genetic variance of depression for women is estimated to lie between 30% and 52%, depending on the severity of the disorder, is in general agreement with previous results which did not account for severity levels. These include two studies by researchers using the Virginia Twin Registry (Kendler et al., 1992, 2001), two studies using the National Swedish Twin Registry (Jansson et al., 2004; Kendler et al., 2006), and one employing the Australian Volunteer Twin Registry (Bierut et al., 1999), which all estimated the genetic component for women to be around 40%. There is also agreement that the equivalent figure for men is considerably less, lying between 21% and 31%. These are additive genetic estimates with the remaining variance due to the random environment and with no shared environmental component. However, little account was taken in these analyses of any contribution due to a possible dominant genetic component. In the case of the first Virginia Twin Registry analysis with 1,033 twin pairs, a 13% dominant component was estimated, and in the Australian study, the AE model was chosen rather than the ADE model for reasons of parsimony. The other studies did not include the ADE model in their analyses.

So, the finding from this study that 25% of the variance is estimated to be due to a dominant component, in addition to the 27% additive component, is a significant factor in understanding the form of the genetic association of depression, and is in contrast to these earlier results.

The minimal contribution found in earlier studies for any dominance component may be partly explained by a lack of statistical power. These studies treated depression as a dichotomous trait using varying DSM and other threshold criteria with different prevalence rates. Analytical studies by Neale, Eaves, and Kendler (1994) have confirmed that the power to detect genetic dominance is low in the classical twin model and this is particularly the case with dichotomous data when the threshold is set at a high level with a low incidence, or prevalence rate. As an example, Neale et al. calculated if the true world variation is 30% additive, 30% dominance, and 40% random environmental, then, in excess of 20,000 twin pairs would be required to reject a false AE model with a power of 80%, if the prevalence rate for the disorder is only 10%. Following their analysis, this study has gained power in two ways: by setting a low initial threshold relating to the incidence of depression, it has resulted in a relatively high overall prevalence rate of 37%, as well as by the gain received from having four ordinal levels rather than being dichotomous in form. According to the Neale et al. analysis, even with these gains, some 3,000 twin pairs would be required for a power of 80% to reject the AE model in favor of the real world ADE model. With 1,449 pairs in this study, the power is only about 50% although examination of Table 1 shows that this has proved sufficient to reject the AE model in favor of the ADE one.

The importance of the dominance contribution can be inferred directly from the depression data where the MZ correlations were found to be more than twice those of the DZ values. This data leads to broad‐sense heritability values of 52% when the severe level is included and 24% when it is excluded using Falconer's formula based on the difference in MZ and DZ correlation values (Falconer, 1965). The higher value of 52% may be compared to that of 58% from the equivalent correlation values reported from the large Swedish National twin study using modified DSM‐IV criteria (Kendler et al., 2006). In this present study, if the contribution of the dominance component is excluded then the narrow‐sense heritability due only to any additive component is 27% when the severe level of depression is included and approximately 0% when it is excluded. The corollary of these estimates is that the milder forms of the disorder are more dependent on lifetime environmental influences than genetic effects.

## 5 Conclusion

The particular contribution of this study is that it has attempted to gain some insight into whether the form and magnitude of the genetic and environmental variance depends on the severity of the disorder using ordinal data. The results suggest that the genetic element of the severe level of depression is largely due to the additive component and that when this depression level is excluded, the origin of the more moderate, broader, forms of the disorder is due to the dominance deviation component combined with a larger random environmental component. If this is correct, then the severe level is dependent on defined homozygosis, which is the presence of certain identical alleles at corresponding multiple loci on homologous chromosomes. The more moderate forms would then be dependent on heterozygosis, or dissimilar alleles at corresponding multiple heterozygotic loci. The mathematical analysis of additive and dominance deviation variance which led to the generation of the classic genetic structural path model has been shown to apply to such multiple loci and also to unequal gene frequencies (Mather & Jinks, 1982; Neale & Cardon, 1992). This is important since the review by Flint and Kendler (2014) suggests that it is likely that a large number of loci, possibly of small effect, are involved in this disorder.

In view of the weakness of the classical twin analysis to reject the AE model in favor of the ADE model, and the fact that this study is limited in power, it is important to replicate these estimates using a larger sample. If it is confirmed that severe forms of female depression are largely associated with the additive genetic component and the more moderate forms with a dominance deviation component plus a larger random environmental variance, then this has implications for the design of GWAS and linkage surveys. Such surveys should be sex specific and recruit those with the severest, more heritable, forms of the disability in order to attempt to detect significant homozygotic genetic loci. In this context, a recent large‐scale study, named CONVERGE, has recruited some 5,000 Chinese women with recurrent major depression in order to reduce such heterogeneity and to identify specific genes through whole‐genome sequencing (CONVERGE Consortium, 2015).

Confirmation of these present results would, in addition, be of clinical importance in confirming that the severe level of the disorder has a greater hereditary component while the milder forms are likely to be more associated with random, lifetime effects. The finding that the shared or family, environmental component is not significant may surprise clinicians but replicates the same conclusion from previous studies (Bierut et al., 1999; Jansson et al., 2004; Kendler et al., 2006). However, Michael Rutter (1997) has warned that low, or negligible, estimates of the shared component do not preclude some early environmental influences on the outcome since twins and siblings may vary in their susceptibility to the vagaries of family upbringing. More research needs to be done to clarify this result and the developmental and lifetime environmental risks associated with depression.

Aside from the above limitation imposed by the number of twin pairs in this study, there is also the question of self‐report reliability and the validity of the questionnaire used. The reliability concern is mitigated to some extent by the checks inherent in the measure. In the case of validity, it would be valuable to repeat this study with an alternative form of ordinal measure.
